# A Systematic Review of the Cost-Effectiveness of Nurse Practitioners and Clinical Nurse Specialists: What Is the Quality of the Evidence?

**DOI:** 10.1155/2014/896587

**Published:** 2014-09-01

**Authors:** Faith Donald, Kelley Kilpatrick, Kim Reid, Nancy Carter, Ruth Martin-Misener, Denise Bryant-Lukosius, Patricia Harbman, Sharon Kaasalainen, Deborah A. Marshall, Renee Charbonneau-Smith, Erin E. Donald, Monique Lloyd, Abigail Wickson-Griffiths, Jennifer Yost, Pamela Baxter, Esther Sangster-Gormley, Pamela Hubley, Célyne Laflamme, Marsha Campbell–Yeo, Sheri Price, Jennifer Boyko, Alba DiCenso

**Affiliations:** ^1^Daphne Cockwell School of Nursing, Ryerson University, 350 Victoria Street, Toronto, ON, Canada M5B 2K3; ^2^Faculty of Nursing, Université de Montreal and Research Centre of Hôpital Maisonneuve-Rosemont, CSA-RC-Aile Bleue-Room F121, 5415 boulevard l'Assomption, Montréal, QC, Canada H1T 2M4; ^3^KJ Research, Rosemere, QC, Canada J7A 4N8; ^4^School of Nursing, McMaster University, 1280 Main Street West, Hamilton, ON, Canada L8S 4L8; ^5^School of Nursing, Dalhousie University, Box 15000, 5869 University Avenue, Halifax, NS, Canada B3H 4R2; ^6^Department of Oncology, McMaster University, 1280 Main Street West, HSC-3N28G, Hamilton, ON, Canada L8S 4L8; ^7^Health Interventions Research Centre, Ryerson University, 350 Victoria Street, Toronto, ON, Canada M5B 2K3; ^8^Department of Community Health Sciences, Faculty of Medicine, University of Calgary, Health Research Innovation Centre, Room 3C56, 3280 Hospital Drive NW, Calgary, AB, Canada T2N 4Z6; ^9^Fraser Health Authority, Suite 400-13450 102nd Avenue, Surrey, BC, Canada V3T 0H1; ^10^International Affairs and Best Practice Guidelines Centre, Registered Nurses' Association of Ontario, 158 Pearl Street, Toronto, ON, Canada M5H 1L3; ^11^School of Nursing, University of Victoria, P.O. Box 1700 STN CSC, Victoria, BC, Canada V8W 2Y2; ^12^The Hospital for Sick Children, Lawrence S. Bloomberg Faculty of Nursing, University of Toronto, 555 University Avenue, Toronto, ON, Canada M5G 1X8; ^13^Primary Health Care Nurse Practitioner Program, School of Nursing, University of Ottawa, 600 Peter Morand Crescent, Suite 101, Ottawa, ON, Canada K1G 5Z3; ^14^Departments of Pediatrics and Psychology and Neurosciences, Dalhousie University, P.O. Box 15000, 5869 University Avenue, Halifax, NS, Canada B3H 4R2; ^15^School of Health Studies, Western University, Health Sciences Building, Room 403, London, ON, Canada N6A 5B9; ^16^Department of Clinical Epidemiology & Biostatistics, McMaster University, 1280 Main Street West, Hamilton, ON, Canada L8S 4L8

## Abstract

*Background*. Improved quality of care and control of healthcare costs are important factors influencing decisions to implement nurse practitioner (NP) and clinical nurse specialist (CNS) roles. *Objective*. To assess the quality of randomized controlled trials (RCTs) evaluating NP and CNS cost-effectiveness (defined broadly to also include studies measuring health resource utilization). *Design*. Systematic review of RCTs of NP and CNS cost-effectiveness reported between 1980 and July 2012. *Results*. 4,397 unique records were reviewed. We included 43 RCTs in six groupings, NP-outpatient (*n* = 11), NP-transition (*n* = 5), NP-inpatient (*n* = 2), CNS-outpatient (*n* = 11), CNS-transition (*n* = 13), and CNS-inpatient (*n* = 1). Internal validity was assessed using the Cochrane risk of bias tool; 18 (42%) studies were at low, 17 (39%) were at moderate, and eight (19%) at high risk of bias. Few studies included detailed descriptions of the education, experience, or role of the NPs or CNSs, affecting external validity. *Conclusions*. We identified 43 RCTs evaluating the cost-effectiveness of NPs and CNSs using criteria that meet current definitions of the roles. Almost half the RCTs were at low risk of bias. Incomplete reporting of study methods and lack of details about NP or CNS education, experience, and role create challenges in consolidating the evidence of the cost-effectiveness of these roles.

## 1. Introduction

Nurse practitioners (NPs) and clinical nurse specialists (CNSs) have practiced for over 50 years in the United States, followed closely by Canada and the United Kingdom, and the roles are increasingly being implemented in other countries [1]. The quest for improved quality of care and control of healthcare costs are important drivers in the decision to implement these roles. We conducted a systematic review to assess the evidence of cost-effectiveness of NP and CNS roles.

## 2. Background 

Both NPs and CNSs are considered advanced practice nurses [2]. NPs are defined as RNs who have additional education in recognized programs, preferably at the graduate level. They demonstrate advanced competencies to practice autonomously and collaboratively to perform assessments, order laboratory and diagnostic tests, diagnose, prescribe medications and treatments, and perform procedures, as authorized by legislation and their regulatory scope of practice [2], as well as performing an advanced nursing role that includes consultation, collaboration, education, research, and leadership. CNSs are registered nurses (RNs) with a graduate degree in nursing who have expertise in a clinical specialty and perform an advanced nursing role that includes practice, consultation, collaboration, education, research, and leadership [3].

NPs and CNSs function in alternative or complementary provider roles. Those working in alternative roles provide similar services to those for whom they are substituting, usually physicians [4]. Those working in complementary roles provide additional services that are intended to complement or extend existing services. The intention of the alternative role is typically to reduce cost or workload or to address workforce shortages while maintaining or improving the quality of care; in contrast, the intention of the complementary role is to improve the quality of care [5].

During the 1970s, the first randomized controlled trials (RCTs) of NPs demonstrated their safety and effectiveness, as well as patient satisfaction with the NP role [6–17]. NPs improved resource utilization and access to care [14, 18–20], increased primary care services in the community [7], and reduced costs [15]. Over the past 30 years, a number of literature reviews and systematic reviews have summarized the findings of studies evaluating NPs [21–25]. The reviews have consistently shown no difference in the health outcomes of patients receiving NP care when compared to patients receiving physician care, but often both quality of care and patient satisfaction are higher with NP care.

Most RCTs of CNS roles have been published since 1980 except one. In 1977, Pozen and colleagues [26] found that the CNS increased the knowledge of heart disease in patients with myocardial infarction resulting in an increased rate of return to work and a reduction in smoking. Literature reviews and systematic reviews of CNSs [25, 27] reveal that CNSs are associated with reductions in hospital length of stay, readmissions, emergency room visits, and costs, as well as improvements in staff nurse knowledge, functional performance, mood state, quality of life, and patient satisfaction.

Study findings are consistent that NPs and CNSs, either in alternative or complementary provider roles, deliver high quality patient care that results in high patient satisfaction. To address a question that often surfaces, “are NPs and CNSs cost-effective?,” we conducted a systematic review of RCTs of NP and CNS cost-effectiveness (defined broadly to also include studies measuring health resource utilization) entitled “*A systematic review of the cost-effectiveness of nurse practitioners and clinical nurse specialists: 1980 to July 2012*.” The purpose of this paper is to report on the methodological strengths and threats to internal and external validity of these RCTs.

## 3. Methods

### 3.1. Eligibility Criteria

We sought RCTs of NP and CNS cost-effectiveness between January 1980 and July 2012. Due to inconsistencies in the use of titles and lack of role clarity for these two roles [28], we developed specific criteria to decide if the role was an NP, a CNS, or an RN in an expanded role. To be deemed an NP, the nurse had to have completed a formal postbaccalaureate or graduate NP education program or be licensed as an NP. To be deemed a CNS, the nurse had to have completed a graduate degree and the role had to be reflective of the CNS role definition. If necessary, we contacted the lead author and/or experts in advanced practice nursing from the country where the study was conducted to determine eligibility.

The principal outcomes of interest in this review were objective measures of health system utilization. These included length of stay, rehospitalization, costs of healthcare (e.g., hospital, professional, and family costs), and health resource use (e.g., diagnostic tests and prescriptions). Because it is important to examine health system utilization in the context of patient and provider outcomes, we also extracted data on all patient (e.g., mortality, morbidity, quality of life, and satisfaction with care) and provider (e.g., quality of care and job satisfaction) outcomes.

Participants were patients of any age receiving care in all types (e.g., teaching and nonteaching, public and private), sizes (e.g., small, medium, and large), and locations (e.g., rural and urban) of hospitals or community settings (e.g., long-term care, primary care, and home care).

Substantive developments since 1980 (e.g., training, payment models, and scope of practice of NPs) have reduced the relevance of pre-1980 studies to modern-day policy. In consultation with a policy advisor, we chose to exclude pre-1980 studies from this review. Studies were also excluded if (1) the NP or CNS education failed to meet our criteria or if we could not contact the author for clarification despite repeated attempts; (2) the NP or CNS was part of a multicomponent or multidisciplinary intervention in which the impact of their contribution could not be isolated from other healthcare providers on the team; (3) the study evaluated a very specific intervention (e.g., cognitive behavioural therapy) that was delivered by an NP or CNS but could be delivered by other clinicians, such as an RN; (4) the control group was also exposed to an NP or CNS during the study; (5) a measure of health system utilization was not included; (6) true randomization was not used (randomization was predictable, for example, assignment by day of hospital admission and alternating assignment).

### 3.2. Search Strategy

A search was conducted to identify all relevant published and unpublished RCTs reported from January 1980 to July 2012. No restrictions were imposed on jurisdiction or language. Medical librarians conducted a comprehensive search of the literature using CINAHL, EMBASE, Global Health, HealthStar, Medline, Allied and Complementary Medicine Database (AMED), Cochrane Library Database of Systematic Reviews and Controlled Trials Register, Database of Abstracts of Reviews of Effects (DARE), Health Economics Evaluation Database (HEED), and Web of Science. Relevant Medical Subject Headings (MeSH) keywords, inclusive suffixes, and search strings formed the search strategy (appendix). In addition, the following methods were used to identify primary studies: handsearching of 16 high-yield journals, checking reference lists of all relevant papers and reviews, contacting authors of an early list of relevant studies, searching personal files, reviewing bibliographies, and searching websites of nursing research and professional organizations and national, provincial/state, and territorial governments.

### 3.3. Study Selection

We uploaded all identified citations to a web-based reference management program (RefWorks) and removed duplicate entries. Two-member teams independently screened titles and abstracts of these citations for relevance using prespecified criteria. Translators assisted with the review of all citations in languages other than French or English. The full-text of a published paper and/or study report was obtained if it appeared to meet the inclusion criteria, if an abstract was unavailable, or if it was not possible to determine relevance from the title and abstract review. In instances where a study was reported in more than one paper, we grouped the study's papers in a constellation and collectively reviewed them. Two-member teams independently screened these full-text papers for eligibility based on the inclusion criteria. Discrepancies were discussed and resolved by consensus. We catalogued all excluded studies and the reason for exclusion. Studies that met eligibility criteria advanced to the quality assessment phase of the review.

### 3.4. Quality Assessment

Two team members (AD and KR) independently assessed the methodological quality of the studies for internal validity and disagreements were resolved through discussion and consensus. The internal validity of each study was assessed using a slightly modified version of the Cochrane risk of bias criteria [29]; modifications to the criteria were three-fold. First, we did not assess for blinding of participants and personnel because the nature of NP and CNS interventions precludes this possibility. Second, outcome assessment and completeness of outcome data were evaluated separately for objective and subjective outcomes within a study. We looked for evidence of key outcomes that would typically be measured for each study's research question [29]. Third, if outcomes had more than 20% missing data, we judged the study to be at high risk of bias for “incomplete outcome data.”

We assessed studies, assigning a high, low, or unclear risk of bias for each of the following eight questions: (1) To avoid selection bias, was the strategy used for random sequence generation likely to produce comparable groups (e.g., random number table, computer random number generator)? (2) To avoid selection bias, was a method used to conceal the allocation sequence so that group allocation could not be foreseen in advance (e.g., sequentially numbered, opaque, sealed envelopes; central allocation office)? (3) To avoid detection bias, was an appropriate method/source used to collect objective (e.g., mortality) measures (e.g., death records, blinding of outcome assessor, trained chart abstracter)? (4) To avoid detection bias, was an appropriate method used to collect subjective (e.g., quality of life) measures (e.g., blinding of outcome assessor; use of reliable, valid, established self-administered questionnaires)? (5) To avoid attrition bias, was outcome data complete for the objective measures (i.e., complete for ≥80% of sample; missing data balanced between groups; missing data imputed using appropriate methods)? (6) To avoid attrition bias, was outcome data complete for the subjective measures (i.e., complete for ≥80% of sample; missing data balanced between groups; missing data imputed using appropriate methods)? (7) To avoid reporting bias, were all outcomes described in the methods section of the study reported in the results and were all key outcomes reported? (8) Were “other” biases detected in the study (e.g., contamination bias in which the control group had exposure to the intervention)?

We sought clarification from 40 of the 43 study authors when there were insufficient details in the paper to determine the risk of bias and we received 28 (70%) responses. An overall risk of bias was assigned to each study as follows: low risk of bias (at risk in 0-1 category), moderate risk of bias (at risk in 2-3 categories), high risk of bias (at risk in 4–6 categories), and very high risk of bias (at risk in 7-8 categories).

External validity refers to the generalization or applicability of the study to other circumstances [30]. To assess external validity, two team members independently assessed the generalizability of the study population, intervention, control, and outcomes (PICO). Disagreements were resolved through discussion and consensus. Historically, RCTs of NPs and CNSs have been criticized because the number evaluated in any study has been small (e.g., one or two NPs) causing concern that those willing to be evaluated may be atypical in training, experience, knowledge, skills, or practice characteristics. We consulted with our policy advisor and together decided that 10 NPs or CNSs either within a single study or across studies combined in meta-analyses would be a reasonable minimum sample necessary to generalize results to similar NP or CNS roles.

As reported in a separate paper, we applied the Quality of Health Economic Studies (QHES) instrument [31–33] to evaluate the economic analyses in each study. The quality of the body of evidence for individual outcomes was evaluated using the Grading of Recommendations Assessment, Development and Evaluation (GRADE) system [34, 35] and GRADEpro software. The results of the GRADE assessments are reported elsewhere [36].

### 3.5. Data Extraction

A trained research assistant (KR) extracted data from each study into a summary table regarding general information (i.e., author, country, setting, language of publication, and publication status), characteristics of the study (design and group allocation), characteristics of the participants (number per group, sex, ages, and health conditions), characteristics of the intervention (number and type of NPs or CNSs, education and training, specific role, and comparison intervention), outcomes (health system, patient, and provider), length of follow-up, proportion followed to study completion, and study findings. If the findings of a single study were reported in two or more papers, they were extracted as one study. Team members checked the accuracy of extractions and discrepancies were resolved through discussion and consensus.

### 3.6. Analysis

Studies were categorized into the following six groupings: NP-outpatient, NP-transition, NP-inpatient, CNS-outpatient, CNS-transition, and CNS-inpatient. In a transition role, the NP or CNS could provide “time-limited services designed to ensure healthcare continuity, avoid preventable poor outcomes among at-risk populations, and promote the safe and timely transfer of patients from one level of care to another or from one type of setting to another” [37, page 747]. Within these groupings, studies were further categorized into alternative or complementary NP or CNS role function.

The strengths and threats to internal and external validity of the included RCTs are summarized narratively, organized by the six groupings identified above.

## 4. Results

### 4.1. Results of the Search

**Figure 1:**
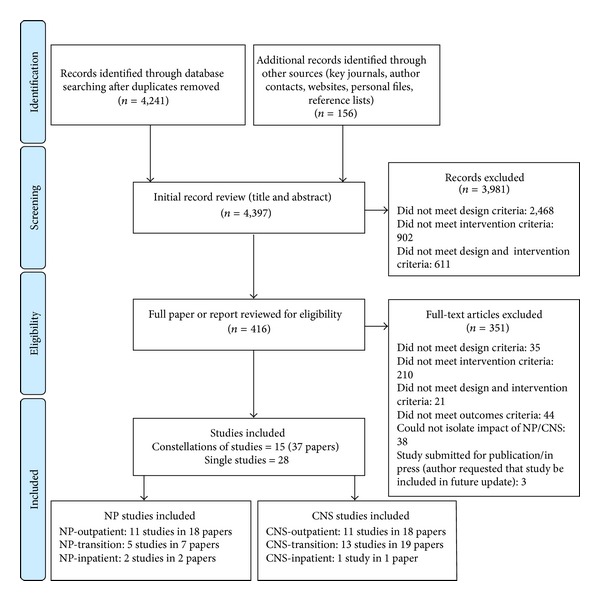
Identification and screening of relevant studies. Flow diagram adapted from Moher et al. [109].

The searches yielded 4,397 unique records of which 3,981 were excluded during title and abstract review. Based on full-text review of the remaining 416 papers, 351 were excluded based on reasons listed in Figure 1. The remaining 65 papers described 43 relevant RCTs (28 studies reported in single papers and 15 studies reported in 37 papers). All studies were published in English. In general, the control intervention was “usual care.”

**Figure 2:**
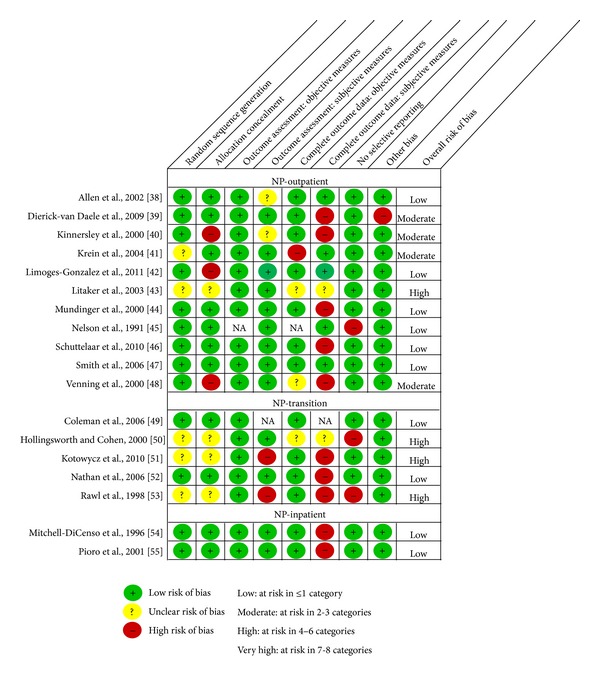
Risk of bias assessment of NP studies (*n* = 18).

**Figure 3:**
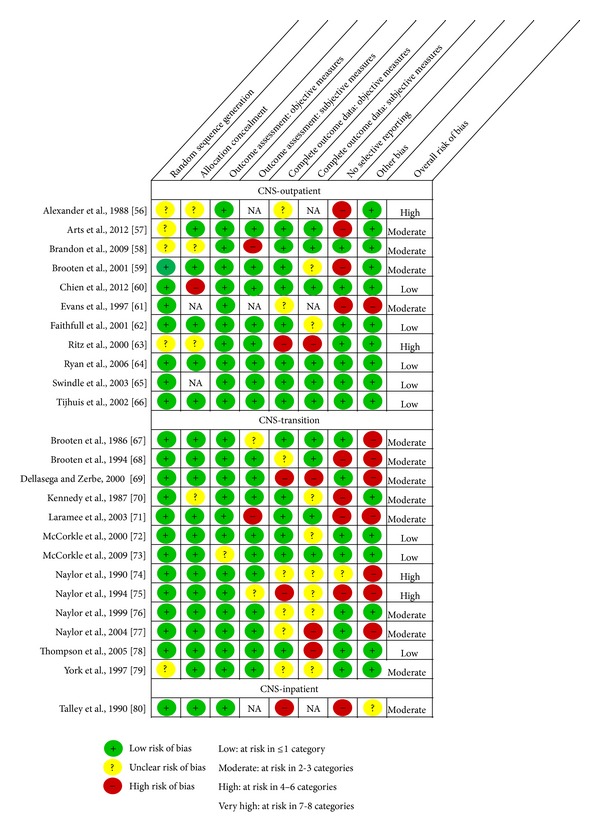
Risk of bias assessment of CNS studies (*n* = 25).

The distribution of the 43 RCTs across groupings was NP-outpatient (*n* = 11), NP-transition (*n* = 5), NP-inpatient (*n* = 2), CNS-outpatient (*n* = 11), CNS-transition (*n* = 13), and CNS-inpatient (*n* = 1). We summarize the results by grouping beginning first with a brief overview of the study characteristics (Tables 1 and 2) followed by a description of threats to internal validity (Figures 2 and 3). Finally, threats to internal and external validity across studies will be described.

### 4.2. Study Characteristics and Internal Threats to Validity

#### 4.2.1. NP-Outpatient Care

Eleven RCTs of NPs in outpatient care [38–48] met our inclusion criteria (Table 1). All but one were published in the year 2000 or later. They were conducted in the United States (*n* = 7), United Kingdom (*n* = 2), or the Netherlands (*n* = 2). Six studies evaluated NPs in alternative provider roles and five in complementary provider roles. The number of NPs ranged from one to 20 in NP alternative provider studies and from one to four in NP complementary provider studies. Some of the trials were quite large with over 1000 patients, while most of the trials examining specific patient populations tended to be much smaller. The studies were conducted at between one and 20 sites.

*Threats to Internal Validity.* Overall, six of the 11 RCTs were judged to be at low risk of bias (in other words, the methods were of high quality), four at moderate, and one at high risk of bias (Figure 2). With regard to selection bias, nine studies used a random sequence generation process that was likely to produce comparable groups; for two studies, we had insufficient information about the sequence generation process to permit judgement, despite contact with one of the authors. Seven trials used an adequate process to conceal allocation so that participants and those enrolling participants could not foresee the group to which the next patient would be assigned. We judged one study as unclear because there was insufficient information and three at high risk of selection bias.

All the RCTs were judged to be at low risk of detection bias with respect to objective outcome measures (e.g., blood levels and medical records abstraction) and all but two trials were assessed at low risk of detection bias for subjective measures because most used established validated self-report instruments (e.g., SF-12 and SF-36). Three trials used blinded assessors for some data collection. Two studies were judged as unclear, one because they used self-reported dietary intake and physical activity which can be subject to recall and social desirability bias and the other because clinicians self-recorded the length of time they spent with each patient.

Seven RCTs were judged to be at low risk of attrition bias for the objective measures; one study reported a follow-up rate less than 80% for a blood cholesterol measure, and two did not report all follow-up rates. The risk of attrition bias for subjective measures was high or unclear for six studies due to failure to report follow-up rates or poor response rates for at least one self- or interviewer-administered questionnaire by last follow-up.

One study was judged at high risk of reporting bias because they did not report any patient outcomes such as child's health status, quality of life, or parent satisfaction in a study of the appropriateness of follow-up care after attendance at an emergency department. We rated one study at high risk of “other” bias because there was substantial baseline imbalance which was not adjusted for in the analyses.

#### 4.2.2. NP-Transition Care

Five RCTs evaluated NPs in a transition role [49–53] (Table 1). Three studies were conducted in the US, one in Canada, and one in the UK. Four of the studies were published in the year 2000 or later. One study evaluated NPs in an alternative provider role and four in complementary provider roles. One or two NPs were evaluated in each study. The number of patients included in the trials ranged from 54 to 750 and they were conducted at between one and 10 sites.

*Threats to Internal Validity.* Overall, two studies were judged to be at low risk of bias and three at high risk of bias (Figure 2). The trials assessed to be at low risk of selection bias used a random number generator that revealed the intervention assignment when a patient was ready for allocation and a computer generated sequence concealed in sequentially numbered, opaque, sealed envelopes. The other three studies provided insufficient information to fully judge random sequence generation and allocation concealment.

All five RCTs were judged to be at low risk of detection bias for objective measures as they used abstraction of hospital administrative records or blinded outcome assessment. With respect to subjective measures, two trials were at high risk of detection bias because patients self-reported their smoking cessation success and the NP, who delivered the intervention, also collected baseline and outcome data from the comparison groups during a guided interview.

All but one trial were at low risk of attrition bias for objective measures as they followed over 80% of participants and this was balanced across comparison groups within each study. With respect to subjective data, three trials scored high for risk of attrition bias due to poor response rates to self- or interviewer-administered questionnaires.

Two studies identified outcomes that they planned to measure but did not report, placing them at risk of reporting bias.

#### 4.2.3. NP-Inpatient Care

Two RCTs of NPs in inpatient settings met our inclusion criteria, both of which evaluated the NP in an alternative provider role [54, 55] (Table 1). One study was conducted in the US and one in Canada. One study was conducted before and one after the year 2000. The number of NPs in the trials ranged from 2.5 to 4.5 full-time equivalent NPs. The number of patients included in the trials ranged from 381 to 821 and each study was conducted at one site.

*Threats to Internal Validity.* Overall, the two studies were judged to be at low risk of bias (Figure 2). Both studies were at low risk of selection bias having used acceptable random sequence generation processes (table of random numbers; computer random number generator) and having concealed allocation through the use of sequentially numbered, sealed, opaque envelopes.

Both were at low risk of detection bias as they relied on medical record and hospital database extraction of objective data such as mortality, medical complications, and length of hospital stay. In cases where study participants completed questionnaires, there were reliable, valid measures such as the SF-36 and the Minnesota Infant Development Inventory (MIDI).

Both studies were judged to be at low risk of attrition bias for the objective measures but at high risk of attrition bias for the subjective measures. While many of the primary objective outcome data were available for all study participants (e.g., mortality, complications, and length of stay), subjective self-report measures often had response rates less than 80%. We judged both studies to be at low risk of reporting bias and other biases.

#### 4.2.4. CNS-Outpatient Care

Eleven RCTs [56–66] addressed the CNS role in delivering outpatient care (Table 2). Six studies were conducted in the US, two in the UK, two in the Netherlands, and one in China. Nine studies were published in the year 2000 or later. Four trials evaluated one to six CNSs in the alternative provider role, while seven trials evaluated one to nine CNSs in the complementary provider role. The number of patients included in the trials ranged from 20 to 643 and the studies were conducted at between one and six sites.

*Threats to Internal Validity.* Overall, five of the eleven studies were assessed at low risk, four at moderate risk, and two at high risk of bias (Figure 3). While seven studies used valid methods to generate the random sequence and were at low risk of selection bias, we judged the remaining four to be at unclear risk of bias because the authors did not include this information in their papers and we did not receive responses to our request for further details. With respect to allocation concealment, five trials were assessed at low risk of selection bias (e.g., central allocation and sealed envelopes) and three at unclear risk of bias because methods were not described. We judged one at high risk of bias because the patients were randomly assigned by the CNS to one of the study groups by drawing the next allocation from an envelope; using this method, it is possible that the drawn assignment could be returned to the envelope and redrawn if allocation was deemed unsuitable. Two studies used cluster randomization and allocation concealment was not applicable as the clusters were all randomized at one time.

All the studies were rated as low in risk of detection bias for objective outcome measures (e.g., mortality and rehospitalization). Of the nine studies that included subjective outcomes, eight were judged at low risk of bias as they used established, validated instruments, or blinded outcome assessment and one was at high risk of bias because the CNS who delivered the intervention also collected data from both groups before and after the intervention via telephone interviews.

With respect to attrition bias, all but three studies were assessed at low risk of bias for objective measures. Two of the studies judged at unclear risk of bias provided insufficient information to assess the completeness of all objective outcome measures and one, judged at high risk of bias, did not have cost data for at least 80% of the study participants.

While seven trials were judged to be at low risk of reporting bias, four were judged at high risk because they did not fully report all the outcomes they collected or did not collect all patient-important outcomes that would have been expected (e.g., patient/parent satisfaction with care and quality of life). Finally, one trial was judged at high risk of “other” bias because they did not adjust for cluster randomization.

#### 4.2.5. CNS-Transition Care

Thirteen RCTs [67–79] evaluated the CNS in the delivery of transition care in the US (*n* = 12) and in the UK (*n* = 1) (Table 2). Seven studies were conducted before the year 2000. All trials evaluated the CNS in a complementary provider role. The studies included between one and seven CNSs. The number of patients included in the trials ranged from 40 to 375 and the studies were conducted at between one and six sites.

*Threats to Internal Validity.* Overall, three of the thirteen trials were at low risk, eight at moderate risk, and two at a high risk of bias (Figure 3). Most trials were not at risk of selection bias. All but one trial used valid methods to generate the random sequence and all but one trial concealed allocation.

All trials were rated at low risk of detection bias for objective measures, except one. In this study, the risk of bias was unclear because healthcare utilization outcomes were based on self-report rather than medical record review data. For subjective measures, two trials were judged to be at unclear risk of detection bias because the validity of their scales was not described, and, in one trial, treatment adherence was based on self-report rather than objective measures, such as pill counts and was assessed at high risk of bias.

For objective measures, six trials had a low risk of attrition bias but, for five trials, the risk was unclear and, for two, it was high. For subjective measures, four trials had a low risk of attrition bias but, for six trials, the risk was unclear and, for three, it was high. For those studies in which it was unclear, the response rates were not specified or data were imputed; for those at high risk of bias, the follow-up rate was less than 80%.

Of the 13 trials, four were at high risk of reporting bias, three of which did not report on all outcomes measured and one of which did not include a measure of health status. One study was at unclear risk of bias because it was unclear if measures reported at baseline should have been reported as outcomes. Finally, seven trials were assessed at high risk of “other” bias because there were baseline differences between the groups for which adjustments were not made.

#### 4.2.6. CNS-Inpatient Care

Only one study, conducted in the US in 1990, evaluated the CNS delivering inpatient care [80] (Table 2). The study examined CNSs in a complementary role. Two CNSs participated in the study, which included 107 patients and was conducted at one site.

*Summary of Threats to Internal Validity.* Overall, the risk of bias for this study was judged as moderate (Figure 3). We judged the study at low risk of selection bias and detection bias. The study, however, was judged to be at high risk of attrition bias because over 20% of patients were dropped from the study after randomization as the intervention they received was changed (e.g., sitters discontinued and control group receiving CNS consultation) resulting in unequal distribution of patients in the two groups.

The study was also at high risk of reporting bias because they did not report whether the CNS and staff nurse intervention influenced patient risk behaviours as intended. Contamination bias was possible because the same staff nurses who received coaching from the CNS for intervention group patient management and for charting nursing observations cared for the control group and might have provided the same patient management and charting strategies for them. Because the associated risk of bias was unknown, we judged this as unclear “other” bias.

#### 4.2.7. Summary

**Figure 4:**
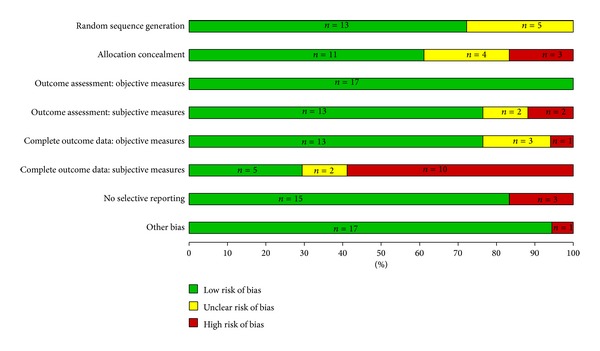
Risk of bias horizontal graph of NP studies (*n* = 18).

**Figure 5:**
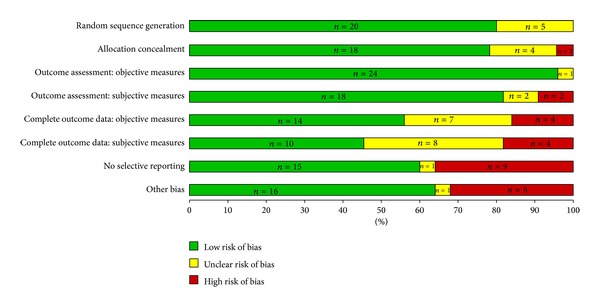
Risk of bias horizontal graph of CNS studies (*n* = 25).

Overall, we assessed that 18 of the 43 trials (42%) were at low risk, 17 (39%) at moderate risk, and 8 (19%) at high risk of overall bias (Figures 2 and 3). No study was judged to be at very high risk of overall bias. Figures 4 and 5 summarize the studies by type of bias. With respect to the NP trials, many studies were at high risk of detection bias with incomplete (<80%) follow-up for subjective outcomes (e.g., self-administered scales). In CNS trials, a number of studies were at high risk of reporting bias because they either did not report on all outcomes measured or did not include a key outcome that we would have expected. A number of studies (especially smaller studies) had baseline differences with no mention of adjusting the analyses to account for these differences.

Some of the potential threats to validity may not in reality be threats, but rather it may be an issue of lack of reporting. There were many instances that we rated categories as “unclear risk of bias” because there was insufficient information in the paper or from the author to permit judgment of low or high risk of bias.

### 4.3. Summary of Threats to External Validity

Of the 43 RCTs, 70% of the studies were conducted in the United States (*n* = 30) and the remainder in four other countries: the United Kingdom (*n* = 6; 14%), The Netherlands (*n* = 4; 9%), Canada (*n* = 2; 5%), and China (*n* = 1; 2%). Given that healthcare systems and NP and CNS education, role implementation, and scope of practice vary internationally, applicability of study findings from one country to another may be compromised.

Some RCTs evaluating NP and CNS roles were conducted across many sites which may enhance generalizability. However, many trials were conducted in single sites, which likely limits the generalizability of study findings.

Of the 43 RCTs, 13 (30%) studies were published prior to the year 2000. Given the substantive progress that has occurred in the development of NP and CNS roles and dynamic changes in healthcare systems internationally, the results of these studies may be less relevant to current-day policy. Although we found a substantial number of eligible RCTs, when broken down by grouping, we identified only one dated RCT of CNSs in the nontransitional care role for inpatient settings. This RCT evaluated two CNSs providing consultation for a small very particular population of medical-surgical patients requiring sitters due to the risk of self-harm or unpredictable behaviour. Similarly, we identified only two RCTs of NPs in the nontransitional care role in inpatient settings, both of which were published over 10 years ago. One study evaluated NPs caring for a homogeneous population of critically ill infants in a Canadian hospital and the other evaluated NPs caring for a heterogeneous population of adults admitted to general medical wards in a US-based hospital. Given the existence of only three fairly dated RCTs of NPs or CNSs in inpatient settings and somewhat specific populations, caution is needed in generalizing these results to NPs and CNSs in other inpatient settings.

Nine (21%) trials were conducted with small numbers of patients (*n* < 100) with specific health conditions. Four trials of NPs in outpatient settings were large with over 1,000 patients. The larger studies with patients experiencing common conditions are more readily generalizable to the general population than smaller trials with patients experiencing a specific condition. However, one of the larger trials [44] limited study entry to poor, non-English speaking Hispanic people which may limit the generalizability of the findings to other patients seeking primary healthcare.

Twenty-seven (63%) of the RCTs evaluated one or two NPs or CNSs, 9 (21%) evaluated three to five, four (9%) evaluated six to nine, and three (7%) evaluated 10 or more all of which were NP-outpatient studies. The small number of NPs and CNSs evaluated in any study raises concern that the results may not be generalizable to colleagues in similar roles. In some cases when study outcomes were similar we were able to combine study findings which increased the number of NPs or CNSs evaluated for that outcome.

About two-thirds of the studies (*n* = 29; 67%) specified that they evaluated experienced NPs or CNSs (i.e., NPs or CNSs who had completed their training at least one year before the evaluation and/or had graduate degrees). Many of the studies did not include information about training and experience. One study posed concern, as it compared novice NPs who had completed a two-year advanced nursing practice graduate degree in the previous two months with general practitioners who had an average of 16 years work experience [39].

Most studies used reliable and valid outcome measures to evaluate patient-important outcomes such as health status, quality of life, and satisfaction with care which strengthens the generalizability of the findings; however, some studies had very short-term follow-up periods (e.g., two weeks after the patient appointment) which may compromise generalizability of study findings over the long term [39, 48, 60].

## 5. Discussion

The purpose of this paper was to report on the methodological strengths and threats to internal and external validity of RCTs of NP and CNS cost-effectiveness. Based on a comprehensive search of the international literature, we identified 43 RCTs, evaluating NPs (*n* = 18) and CNSs (*n* = 25). While 43 RCTs sound like a large number of evaluations of NPs and CNSs, categorizing the studies by NP or CNS role (i.e., alternative or complementary) and by setting (i.e., outpatient, transition, or inpatient) reveals the areas where further research is still required. For example, we found only one RCT of the CNS in a nontransitional role in the inpatient setting and only two RCTs of the NP in a nontransitional role in the inpatient settings, both of which were alternative provider roles.

Of the 43 RCTs, 70% (*n* = 30) were conducted in the United States with far fewer conducted in four other countries (Canada, China, The Netherlands, and United Kingdom). In 2011, Newhouse et al. conducted a systematic review of the effectiveness of NPs and CNSs [25]. They chose to restrict the review to studies conducted in the United States to enhance the applicability of study findings to the United States healthcare system. A recent systematic review that also includes studies conducted outside the United States has not been conducted, to our knowledge. Therefore, we chose to broaden our search to include international studies in order to learn more about where NP and CNS role evaluations have been conducted and how the roles are being enacted globally.

### 5.1. Internal Validity

Our assessment of the risk of bias revealed that about two-fifths (*n* = 18; 42%) of the 43 studies were at low risk of bias, close to the same number (*n* = 17; 39%) were at moderate risk of bias, and about one-fifth (*n* = 8; 19%) at high risk of bias. When examined by date, 31% of the 13 RCTs published before the year 2000 were at high risk of bias compared to 13% of the 30 RCTs published in or after the year 2000 that were at high risk of bias which may mean that study validity is improving over time.

In many cases it was unclear if the authors met the risk of bias criteria because the required information was not reported in the paper. Consequently, we rated a large number of categories as “unclear risk of bias.” To permit complete and accurate assessment of risk of bias, researchers are encouraged to use a guide such as the Cochrane risk of bias criteria [29] when planning and reporting future studies.

Researchers reporting RCTs may also find the following recommendations helpful. A clear brief description of the sequence generation (e.g., random number table; computer random number generator) is needed to allow the reader to determine if the process should provide comparable groups [29]. A description of allocation concealment (e.g., sequentially numbered, opaque, and sealed envelope) is important for the reader to determine if allocation to groups could be manipulated. While blinding of participants is not possible in a study incorporating NPs or CNSs, a description of procedures used to blind outcome assessors and/or the description of valid outcome measures is needed to assess the quality of the study. Completeness of outcome data for each outcome measure and group, including the description of missing data and details of all participants excluded, lost to follow-up (e.g., dropped out of study or died), or reincluded at each stage, also needs to be reported. If researchers do not report outcomes that were measured or key outcomes that would be expected, a clear description is needed of the reasons for failing to report the outcome. A description of how any “other” biases were managed that threaten the quality of the study should also be reported. More detailed recommendations for reporting RCTs can be found in the Consolidated Standards of Reporting Trials (CONSORT) 2010 Statement [81, 82]. When authors are faced with cutting back on the number of words in a publication, a suggestion is to reduce the introductory sections to provide sufficient space to describe in detail the strategies used to prevent or minimize threats to internal validity.

### 5.2. External Validity

As others have found [5, 24], a challenge in conducting this systematic review was determining the fidelity of the intervention. The definition of the role and the education, training, and experience of the NPs or CNSs were often inadequately described or missing. When we contacted authors for this information, we found that some studies were conducted with RNs who had received as little as a few weeks of training or one course and were then called “NPs.”

Over half (*n* = 27; 63%) of the 43 studies evaluated only one or two NPs or CNSs and only three trials, all of NPs in outpatient settings, evaluated 10 or more. Approximately two-thirds of the studies (*n* = 29; 67%) evaluated experienced NPs or CNSs. Researchers are encouraged to include a detailed description of the NPs or CNSs being evaluated in their study (role in the context of an internationally accepted definition [2], education, experience in the role, and training for the specific intervention if applicable). Similar information should be provided for comparison providers. Furthermore, evaluations of these roles should not be initiated while the NPs or CNSs are still novices but rather when they have had sufficient experience in their role (i.e., at least 12 months). Challenging as it is, researchers are encouraged to plan multisite studies, to increase the number of NPs or CNSs evaluated, to increase the number of patients enrolled in the study, and to account for variations in practice to enhance the generalizability of study findings.

### 5.3. Strengths and Limitations

Restricting this review to RCTs may be viewed as a strength or limitation, depending on the perspective of the reader. Health service settings are complex and research is confounded by multiple variables that challenge the ability to evaluate the effectiveness of an intervention, such as NP and CNS roles. When feasible, randomization of participants to intervention and control groups is considered the optimal design to control known and unknown complexities and confounding variables [83–86]. Therefore, we chose to limit our review to RCTs. The quality of evidence in this review demonstrates that it is feasible to conduct well-designed RCTs to evaluate the effectiveness of NP and CNS roles in a variety of settings, remuneration mechanisms, and patient populations.

Strengths of our review include use of numerous strategies to identify all RCTs in any language (published or unpublished) that met our inclusion criteria, contact with authors and international expert advisors when it was unclear whether a study met our inclusion criteria, use of current education and credentialing criteria to verify that the trial was indeed evaluating an NP or CNS, use of duplicate assessment by independent reviewers and a consensus process for every stage of the review, use of an internationally recognized and established tool to assess the overall risk of bias of each trial and contact with authors when additional information was required to make our assessment, use of an established tool (Quality of Health Economic Studies) to evaluate the health economic analysis in each study, use of GRADE to evaluate outcome-specific quality of evidence, consideration of external as well as internal validity, grouping of trials by type (NP or CNS), setting (inpatient, transition, or outpatient), and role (alternative or complementary), and conducting meta-analyses whenever possible.

In future publications, we will summarize our assessment of the quality of the economic analyses of each RCT and outcome-specific quality of evidence using GRADE for each of the six groupings.

With respect to limitations, despite our attempts to identify all relevant RCTs, we may have missed some relevant studies or included some that do not meet our criteria based on author responses, advisor advice, or our interpretation of the description of the education or role. With respect to generalizability, we did not use a specific tool to assess threats to external validity but did consider the country and year of publication, number of NPs or CNSs in the study, the number of settings, and characteristics of the population, setting, intervention, and outcomes. We do not know how the exclusion of observational studies that investigate the effectiveness of NP and CNS roles may have influenced our findings [86].

## 6. Conclusions

This paper builds on the body of knowledge regarding quality of RCTs of NP and CNS cost-effectiveness (defined broadly to also include studies measuring health resource utilization). We have used an international lens and inclusion criteria that meet today's definitions of the NP and CNS roles. While almost half the RCTs were found to be at low risk of bias, incomplete reporting of study methods and lack of details about NP and CNS education, experience, and roles make it difficult to fully evaluate the internal and external validity of studies of these roles. Future studies that adhere to current standards for internal validity,such as Cochrane risk of bias [29], CONSORT [81, 82], and GRADE [34, 35], will contribute to a stronger body of evidence to address policy makers' questions regarding the cost-effectiveness of NP and CNS roles.

## Figures and Tables

**Table 1:** Summary of NP study characteristics.

Author, year, and country (additional publications)	Study objective (number analyzed)	Participants	Intervention(NP role)	Number of sites	Number of NPs experience and training
NP in outpatient setting (*n* = 11)					
Allen, 2002, US [38] (Paez and Allen, 2006) [87]	Compare NP plus usual care (*n* = 115) to usual care (*n* = 113) in the management of blood lipids in patients with CHD	228 adults with hypercholesterolemia and CHD who were hospitalized for CABG or PCI	NP counseled on lipid management and lifestyle changes and had permission to prescribe (complementary role)	1	1 NP Education and experience were not reported

Dierick-van Daele, 2009, NL [39] (Dierick-van Daele et al., 2010) [88]	Compare NP (*n* = 817) and GP (*n* = 684) in the provision of primary care	1501 patients attending a primary care appointment for common complaints	NP saw patients at first point of contact; a GP was required to sign off all prescriptions (alternative role)	15	12 NPs All completed a 2-year ANP MSc in previous 2 months

Kinnersley, 2000, UK [40]	Compare NP (*n* = 652) and GP (*n* = 716) in the provision of primary care	1465 (1368 analyzed) patients seeking a same-day appointment	NP saw patients at first point of contact; a GP was required to sign off all prescriptions (alternative role)	10	10 NPs All completed an NP diploma at least 1 year previously

Krein, 2004, US [41]	Compare NP plus usual care (*n* = 123) and usual care (*n* = 123) in the management of type 2 diabetes	246 adults with type 2 diabetes and poor glycemic control	NP followed the Chronic Care Model in helping patients to manage glucose levels; PCPs were required to approve medication changes (complementary role)	2	2 NPs 2-day training session; other education and experience were not reported

Limoges-Gonzalez, 2011, US [42]	Compare gastroenterology NP (*n* = 50) to gastroenterologists (*n* = 100) in screening colonoscopies	150 average risk patients ≥50 yrs who were referred for a screening colonoscopy	NP performed the colonoscopy under the same conditions as medical doctors and polypectomies were performed by the NP independently (alternative role)	1	1 NP Intensive training followed by 2 years of practice

Litaker, 2003, US [43]	Compare NP plus usual care (*n* = 79) and usual PCP care (*n* = 78) in the management of patients with hypertension and diabetes	157 adult patients with mild-moderate hypertension and NIDDM without end-organ complications	NP saw patients at first point of contact and provided telephonic and in-office management; permission to prescribe was not reported (complementary role)	1	1 NP∗ Specific training for the management of diabetes and hypertension

Mundinger, 2000, US [44] (Lenz et al., 2002; Lenz et al., 2004) [89, 90]	Compare NP (*n* = 1181) and physician (*n* = 800) ongoing primary care	1981 ED or urgent care adult patients with no regular source of care	NP saw patients at first point of contact and had authority to prescribe (alternative role)	5	7 PTE NPs School of nursing faculty members with specialties in adult primary care

Nelson, 1991, US [45]	Compare pediatric NP telephone support plus usual care (*n* = 91) to usual care (*n* = 93) of parents after an ED visit for their child's acute illness	190 (184 analyzed) outpatient children (<8 yrs) who attended the ED for an acute infectious or allergic condition	NP made telephone contact with parent(s) after discharge, provided education and treatment review, answered questions, and facilitated communication between family and PCP; permission to prescribe was not reported (complementary role)	2	2 NPs Education and experience were not reported

Schuttelaar, 2010, NL [46] (Schuttelaar et al., 2011) [91]	Compare NP (*n* = 81) and dermatologist (*n* = 79) care of children with eczema	160 children with atopic dermatitis who were newly referred by their GP or paediatrician	NP provided the same services as the dermatologist and was able to prescribe independently (alternative role)	1	1 NP ANP masters prepared with 3-year experience in dermatology

Smith, 2006, US [47] (Lyles et al., 2003; Luo et al., 2007) [92, 93]	Compare NP plus usual care (*n* = 101) and usual care (*n* = 105) in the management of patients with medically unexplained symptoms	206 patients (18–65 years) with medically unexplained symptoms and high utilization of primary care services	NP coordinated and managed care over a minimum of 12 scheduled visits over a year and telephone contact between visits (complementary role)	3	4 NPs Certified with 84 hours of special training, no prior experience in mental health

Venning, 2000, UK [48]	Compare NP (*n* = 651) and GP (*n* = 665) primary care of patients seeking same day consultations	1316 patients of all ages	NP saw patients at first point of contact; a GP was required to sign off all prescriptions (alternative role)	20	20 NPs Diploma, BSc, or MSc prepared with 1–5 years as an NP

NP in transition role (*n* = 5)					
Coleman, 2006, US [49] (Parry et al., 2003) [94]	Compare geriatric NP plus usual care (*n* = 379) and usual care (*n* = 371) of older patients with complex care needs	750 chronically ill, community-dwelling, local, older adults (≥65 yrs) admitted to hospital for 1 of 11 nonpsychiatric conditions	NP met with patient in hospital and made a home visit and telephone calls after discharge; patients transferred to a skilled nursing facility were telephoned or visited at least weekly (complementary role)	10	2 NPs∗ Experienced geriatric NPs who were skilled in patient education and advocacy

Hollingsworth 2000, US [50]	Compare NP-facilitated early discharge, follow-up care plus usual care (*n* = 54) and usual care (*n* = 59) of women who have had an abdominal hysterectomy	113 women (≥21 yrs) undergoing abdominal hysterectomy for nononcologic indications	NPs had contact with patient in hospital, encouraged early discharge, made home visits and telephone calls, and were available for patients and families by telephone (complementary role)	1	2 NPs (1 FTE and 1 PTE) Masters prepared

Kotowycz, 2010, CAN [51]	Compare NP-facilitated early discharge, follow-up care plus usual care (*n* = 27) and usual care (*n* = 27) of patients with low-risk STEMI	54 low-risk (Zwolle Primary PCI Index ≤3) STEMI patients treated with primary or rescue PCI.	NP saw patients before and after discharge and provided education and appointment reminders; permission to prescribe was not reported (complementary role)	1	1 NP∗ Education and experience were not reported

Nathan, 2006, UK [52]	Compare respiratory specialist NP (*n* = 78) and respiratory doctor (*n* = 76) in the provision of follow-up care to acute asthma patients	154 acute asthma patients (>16 yrs) discharged from hospital. Those with COPD were excluded.	NP saw outpatients after discharge and for follow-up appointments; NP prescribed independently according to a patient group directive (alternative role)	1	1 NP Masters prepared with specialist training in acute asthma management

Rawl, 1998, US [53] (Easton et al., 1995) [95]	Compare NP postdischarge follow-up plus usual care (*n* = 49) and usual care (*n* = 51) of rehabilitation patients with long-term disabilities	100 rehabilitation patients (≥18 yrs), who were not confined to their home	NP contacted patients before discharge and in the rehabilitation clinic, in their home, and by telephone after discharge (complementary role)	1	1 NP Certified in rehabilitation, 10-year experience

NP in inpatient setting (*n* = 2)					
Mitchell-DiCenso, 1996, CAN [54]	Compare NP (*n* = 414) and pediatric resident teams (*n* = 407) in neonatal intensive care	821 neonates admitted to the neonatal intensive care unit.	NP team assumed primary responsibility for neonates (alternative role)	1	4.5 FTE NPs All graduates of a 16-month Masters program

Pioro, 2001, US [55]	Compare NP (*n* = 193) and house staff (*n* = 188) in care of general medical patients	381 adult general medical patients	NPs provided many of the same services delivered by traditional house staff (alternative role)	1	2.5 FTE NPs Experience and training were not described

ANP: advanced nurse practitioner; BSc: Bachelor of Science; CABG: coronary artery bypass surgery; CAN: Canada; CHD: coronary heart disease; COPD: chronic obstructive pulmonary disease; GP: general practitioner; ED: emergency department; FTE: full-time equivalent; MSc: Master of Science; NIDDM: non-insulin dependent diabetes mellitus; NL: The Netherlands; NP: nurse practitioner; PCI: percutaneous coronary intervention; PCP: primary care provider; PTE: part time equivalent; STEMI: ST-elevation myocardial infarction; UK: United Kingdom; US: United States.

∗Data provided by author.

**Table 2:** Summary of CNS study characteristics.

Author, year, and country (additional publications)	Study objective (number analyzed)	Participants	Intervention (CNS role)	Number of sites	Number of CNSs experience and training
CNS in outpatient setting (*n* = 11)					
Alexander, 1988, US	Compare CNS (*n* = 11) and usual primary continuity care (*n* = 10) of poorly controlled noncompliant asthmatic children	21 asthmatic children (15 months to 13 years) from low-income families who used the ED as their primary care source	CNS promoted self-care based on the Orem Self-Care Nursing Model; permission to prescribe was not described (alternative role)	1	1 CNS Education and experience were not reported

Arts, 2012, NL	Compare CNS (*n* = 169) and physician (*n* = 168) care and cost-effectiveness in the treatment of patients with diabetes	337 patients with diabetes treated in a hospital-based setting. All required insulin treatment or oral blood-glucose medication and had inadequate regulation of blood glucose, blood pressure, or lipids	CNS managed diabetes patients in same way as the physicians, including diabetes-related clinical admissions; referrals to specialist care required a physician (alternative role)	1	4 CNSs Doctoral or Masters prepared with extensive experience in diabetes care

Brandon, 2009, US	Compare CNS (*n* = 10) and usual care (*n* = 10) of patients with HF	20 adult patients living with HF for >6 months who were capable of self-care	CNS provided education, care management and medication adherence advice, and patient support; permission to prescribe was not reported (complementary role)	1	1 CNS Masters prepared∗ Student practicum under cardiologist supervision plus 10-year experience in intensive and coronary care

Brooten, 2001, US	Compare CNS (*n* = 85 mother; 94 infants) and usual care (*n* = 88 mothers; 100 infants) of high-risk pregnant women	173 pregnant women at high risk due to gestational or pregestational diabetes mellitus, chronic hypertension, or preterm labour with 194 infants	CNS provided prenatal monitoring, assessment, education, counseling, and community referrals; medication regimens were adjusted after physician consultation (complementary role)	1	3 CNS Masters prepared specializing in high-risk pregnancies and infants (experience not reported)

Chien, 2012, China	Compare psychiatric CNS (*n* = 39) and usual care (*n* = 40) of patients with psychiatric symptoms	79 referred adult (18–49 yrs) patients with first-episode, moderately severe psychiatric symptoms who were at low risk of self-harm or violence	CNS provided 6 sessions of assessment, support system design, coordination of care, and education in symptom management; permission to prescribe was not reported (complementary role)	1	1 CNS Masters prepared∗ with training in psychosocial interventions for patients with mental health problems (experience not reported)

Evans, 1997, US (Strumpf et al., 1992; Patterson et al., 1995; Siegler et al., 1997; Capezuti et al., 1998) [96–99]	Compare gerontologic CNS education (*n* = 152), CNS education plus consultation (*n* = 127) and neither education nor consultation (*n* = 184) in the use of physical restraints in nursing homes	643 (463 analyzed) residents (>60 yrs) from 3 nursing homes	CNS education involved ten 30-minute sessions addressing issues surrounding restraint use; CNS consultation involved 12 hours/week of unit-based consultation for residents with clinically challenging behaviour (complementary role)	3	1 CNS Masters prepared (experience not reported)

Faithfull, 2001, UK	Compare CNS (*n* = 58) and usual care (*n* = 57) of men treated with radical radiotherapy for prostate and bladder cancer	115 men undergoing radical (>60 Gy) radiotherapy for prostate or bladder cancer	CNS made initial assessments, had open access clinics during therapy, and made posttherapy telephone contacts; permission to prescribe was not reported (alternative role)	1	1 CNS Masters prepared with expertise in radiotherapy toxicity management∗ (experience not reported)

Ritz, 2000, US	Compare CNS (*n* = 106) and usual care (*n* = 104) of breast cancer patients	210 women with newly diagnosed breast cancer (30–85 years) who were referred by their physician and were cared for within the system	CNS provided assessments, information, support, and coordination of care; permission to prescribe was not described (complementary role)	1	2 CNSs Masters prepared∗ (experience not reported)

Ryan, 2006, UK	Compare rheumatologic CNS plus usual care (*n* = 36) and usual care (*n* = 35) of patients with rheumatoid arthritis	71 patients with diagnosed rheumatoid arthritis who were beginning new disease modifying antirheumatic drugs	CNS provided the same service as the outpatient clinic nurse with addition of assessment and referral responsibilities; permission to prescribe was not reported (complementary role)	1	1 CNS Doctoral preparation with 16-year experience in rheumatology∗

Swindle, 2003, US	Compare mental health CNS (*n* = 134) and physician care (*n* = 134) of veterans with depression	268 new patients with PRIME-MD depression diagnosis	CNS contacted patients by telephone or visits, while the CNS recommended antidepressant medication and changes to type and dose; permission to prescribe was not reported (complementary role)	2	9 CNSs 5 had cognitive behavioral treatment training; 9–23-year experience treating depression

Tijhuis, 2002, NL (Tijhuis et al., 2003; Tijhuis et al., 2003; van Den Hout et al., 2003)[100–102]	Compare CNS outpatient care (*n* = 71), inpatient care (*n* = 71), and day-patient care (*n* = 68) of patients with rheumatoid arthritis	210 rheumatoid arthritis patients with increasing functional limitations	CNS provided information, referrals, and hardware prescriptions; CNS did not have permission to prescribe or change drugs (alternative role)	6	6 CNSs Education and experience were not reported

CNS in transition role (*n* = 13)					
Brooten, 1986, US	Compare perinatal CNS-care (*n* = 36 mothers; 39 infants) and usual care (*n* = 36 mothers; 40 infants) of very-low-birth weight infants	72 mothers and 79 very-low-birth weight infants (≤1500 g)	CNS contacted parent(s) during infant hospitalization and made home visits and telephone contact; permission to prescribe was not reported (complementary role)	1	3 CNSs (1 FTE; 2 PTE) Masters prepared in perinatal and neonatal nursing

Brooten, 1994, US	Compare CNS plus usual care (*n* = 61) and usual care (*n* = 61) of high risk postpartum women	122 postpartum women who had received an unplanned caesarean delivery	CNS provided comprehensive in hospital and follow-up care with postdischarge home visits and telephone calls (complementary role)	1	3 CNSs∗ Education and experience were not reported

Dellasega, 2000, US (Dellasega and Zerbe, 2002) [103]	Compare CNS plus usual care (*n* = 69 patients; 34 caregivers) and usual care (*n* = 71 patients; 31 caregivers) of elderly frail discharged patients	140 elderly patients who were scheduled to be discharged home, were cognitively frail and/or functionally impaired, or were a complex case (plus 65 caregivers)	CNS or NP visited patient before discharge and after discharge; additional telephone calls or visits were initiated as needed (complementary role)	3∗	2 CNSs and 2 NPs Education and experience were not reported

Kennedy, 1987, US (Neidlinger et al., 1987) [104]	Compare gerontologic CNS plus usual care (*n* = 39) and usual care *n* = 41) of elderly patients admitted to nonintensive care units	80 consecutive elderly patients (≥75 yrs) admitted to nonintensive care units who were expected to stay ≥72 hours	CNS met patients, family, and care providers in hospital and again just prior to discharge; permission to prescribe was not reported (complementary role)	1	1 CNS Masters prepared with additional geriatric knowledge and skills

Laramee, 2003, US	Compare CHF CNS plus usual care (*n* = 141) and usual care (*n* = 146) in the management of HF patients admitted to hospital	287 patients at risk of early readmission who had been admitted to hospital for primary or secondary CHF, left ventricular dysfunction <40%, or radiologic evidence of pulmonary oedema	CNS visited patients daily in hospital and made postdischarge telephone contacts (complementary role)	1	1 CNS Masters prepared with 18-year experience in critical care and cardiology

McCorkle, 2000, US (Jepson et al., 1999) [105]	Compare CNS plus usual care (*n* = 190) and usual care (*n* = 185) of older postsurgical cancer patients	375 older (60–92 yrs) newly diagnosed solid-tumor cancer patients discharged after surgery to their home	CNS contacted patients after discharge and made home visits and telephone contacts (complementary role)	1	7 CNSs∗ Completed 2-year program in oncology

McCorkle, 2009, US (McCorkle et al., 2011) [106]	Compare oncology CNS plus usual care (*n* = 63) and usual care (*n* = 60) of women recovering from gynecological cancer surgery	149 (123 analyzed) women (≥21 yrs) with suspected ovarian cancer recovering from gynaecological cancer surgery and undergoing chemotherapy	CNS provided tailored specialized care through 18 postdischarge patient contacts (complementary role)	2	1 CNS and 4 NPs∗ Education and experience were not reported

Naylor, 1990, US	Compare CNS plus usual care (*n* = 20) and usual care (*n* = 20) of elderly patients admitted to hospital	40 English speaking inpatients (≥70 years) who had been admitted to hospital from home.	CNS contacted patients in hospital, implemented the discharge plan, and contacted patients after discharge while coordinating with PCP and providing telephone outreach (complementary role)	1	2 PTE CNSs Masters prepared

Naylor, 1994, US	Compare gerontologic CNS plus usual care (*n* = 140) and usual care (*n* = 136) of elderly patients admitted to hospital	276 English speaking inpatients (≥70 years) admitted from their homes: medical (CHF and angina/MI) and surgical (CABG and CVR) patients	CNS contacted patient in hospital, made postdischarge visits, and was available 7 days/week during hospitalization and after discharge (complementary role)	1	2 PTE CNSs Masters prepared with at least one year experience as a specialist

Naylor, 1999, US (Naylor and McCauley, 1999) [107]	Compare gerontologic CNS plus usual care (*n* = 177) and usual care (*n* = 186) of elderly patients admitted to hospital	363 hospitalized elderly patients (≥65 yrs) admitted to hospital from home who were at risk of readmission	CNS contacted patient in hospital, made home visits and weekly telephone contacts, and individualized patient management; permission to prescribe was not reported (complementary role)	2	5 PTE CNSs Masters prepared with a mean of 6.5 years postdegree experience

Naylor, 2004, US (McCauley et al., 2006) [108]	Compare CNS plus usual care (*n* = 118) and usual care (*n* = 121) of elderly patients hospitalized with HF	239 HF patients ( ≥65 years) admitted to study hospitals from their homes	CNS contacted patients in hospital and after discharge and provided discharge planning, assessments, education, and development and implementation of care goals (complementary role)	6	3 CNSs Masters prepared with specialized training in managing elderly HF patients

Thompson, 2005, UK	Compare CNS plus usual care (*n* = 58) and usual care (*n* = 48) of patients admitted to hospital for HF	106 patients with acute admissions to hospital for CHF and left ventricular ejection fraction ≤45%, who were discharged home	CNS provided clinic and home-based care within 10 days of discharge; permission to prescribe was not reported (complementary role)	2	2 CNSs Postgraduate education with HF management experience

York, 1997, US	Compare perinatal CNS-facilitated early discharge plus usual care (*n* = 44 mothers; 42 infants) and usual care (*n* = 52 mothers; 51 infants) of high-risk pregnant women	96 high-risk pregnant women with either diabetes or hypertension during pregnancy	CNS provided in hospital and postdischarge follow-up care; permission to prescribe was not reported (complementary role)	1	1 CNS Masters prepared

CNS in inpatient setting (*n* = 1)					
Talley, 1990, US	Compare psychiatric liaison CNS consultation (*n* = 47) and no consultation (*n* = 60) for nursing care and the use of sitters	107 acute care patients who had been assigned lay sitters primarily because of a danger of “harm to self” or “generally unpredictable” behaviour	CNS provided individualized consultations to patients, nursing staff, and sitters sometimes on multiple occasions; permission to prescribe was not reported (complementary role)	1	2 CNSs Education and experience were not reported

ANP: advanced nurse practitioner; CABG: coronary artery bypass graft; CHF: congestive heart failure; CNS: clinical nurse specialist; CVR: cardiovascular recovery; GP: general practitioner; ED: emergency department; HF: heart failure; FTE: full-time equivalent; Gy: gray (unit of absorbed radiation); MI: myocardial infarction; MSc: Master of Science; NL: The Netherlands; NP: nurse practitioner; PCP: primary care provider; PRIME-MD: primary care evaluation of mental disorders; PTE: part time equivalent; UK: United Kingdom; US: United States.

∗Data provided by author.

## Appendix

### Electronic Database Search Strategies

*Database*: CINAHL
*Data range*: 1981 to July 31, 2012
*Results*: 713
# Query
S56 S53 and S55
S55 S20 or S21 or S25
S54 S26 and S53
S53 S50 or S51 or S52
S52 S47 or S48 or S49
S51 S37 or S38 or S39 or S40 or S41 or S42 or S43 or S44 or S45 or S46
S50 S27 or S28 or S29 or S30 or S31 or S32 or S33 or S34 or S35 or S36
S49 placebo * N4 control *
S48 placebo * N4 trial *
S47 placebo *
S46 (MH “Placebos”)
S45 tripl * N25 mask *
S44 tripl * N25 blind *
S43 doubl * N25 mask *
S42 doubl * N25 blind *
S41 singl * N25 mask *
S40 singl * N25 blind *
S39 PT clinical trial
S38 (MH “Double-Blind Studies”)
S37 double-blind method *
S36 single-blind method *
S35 (MH “Single-Blind Studies”)
S34 experimental trial *
S33 (MH “Clinical Trials+”)
S32 controlled clinical trial *
S31 randomi?ed experimental trial *
S30 random * allocat *
S29 (MH “Random Assignment”)
S28 rct *
S27 randomi?ed controlled trial
S26 S20 or S24 or S25
S25 nurs * led
S24 S11 or S12 or S13 or S16 or S17 or S18 or S19
S23 S1 or S2 or S3 or S4 or S5 or S6 or S7 or S8 or S9 or S10 or S11 or S12 or S13 or S14or S15 or S16 or S17 or S18 or S19 or S20 or S21
S22 S1 or S2 or S3 or S4 or S5 or S6 or S7 or S8 or S9 or S10 or S11 or S12 or S13 or S14 or S15 or S16 or S17 or S18 or S19 or S20 or S21
S21 S11 or S12 or S16 or S17 or S18 or S19
S20 S1 or S2 or S3 or S4 or S5 or S6 or S7 or S8 or S9 or S10
S19 (MH “Nurse Anesthetists”)
S18 nurse anesthetist *
S17 nurse anaesthetist *
S16 nurse clinician *
S15 NP
S14 np
S13 “np” and nurse *
S12 CNS and nurse *
S11 specialist nurse *
S10 nurse specialist *
S9 nurse practitioner *
S8 (MH “Nurse Practitioners+”)
S7 clinical nurse specialist *
S6 (MH “Clinical Nurse Specialists”)
S5 apn
S4 advanced practice nurs *
S3 (MH “Advanced Practice Nurses+”)
S2 advanced nursing practice *
S1 (MH “Advanced Nursing Practice+”)

*Database*: EMBASE
*Date*: 1980 to July 31, 2012
*Results*: 1552
1. advanced nursing practice *.mp.
2. advanced practice nurs *.mp.
3. apn.mp.
4. clinical nurse specialist *.mp.
5. (cns and nurs *).mp.
6. exp advanced practice nurse/
7. nurse practitioner *.mp.
8. (np and nurse *).mp.
9. nurse specialist *.mp.
10. specialist nurse *.mp.
11. nurse clinician *.mp.
12. nurse an?esthetist *.mp.
13. nurs * led.mp.
14. or/1-13
15. randomized controlled trial/
16. randomi?ed controlled trial *.mp.
17. rct.mp.
18. randomi?ed controlled trial.pt.
19. randomized controlled trial.pt.
20. RANDOMIZATION/
21. random * allocat *.mp.
22. randomi?ed experimental trial *.mp.
23. controlled clinical trial/
24. controlled clinical tria *l.mp.
25. clinical trial/
26. clinical trial *.mp.
27. clinical trial *.pt.
28. experimental trial *.mp.
29. single blind procedure/
30. double blind procedure/
31. triple blind procedure/
32. ((singl * or doubl * or tripl * or trebl *) adj25 (blind * or mask *)).mp.
33. (placebo * or (placebo * adj4 (trial * or control *))).mp.
34. or/15-33
35. 14 and 34

*Database*: Global Health
*Date*: 1973 to July 31, 2012
*Results*: 38
1. advanced nursing practice *.mp.
2. advanced practice nurs *.mp.
3. apn.mp.
4. clinical nurse specialist *.mp.
5. (cns and nurs *).mp.
6. advanced practice nurse/
7. nurse practitioner *.mp.
8. (np and nurse *).mp.
9. nurse specialist *.mp.
10. specialist nurse *.mp.
11. nurse clinician *.mp.
12. nurse an?esthetist *.mp.
13. nurs * led.mp.
14. or/1-13
15. randomized controlled trial/
16. randomi?ed controlled trial *.mp.
17. rct.mp.
18. randomi?ed controlled trial.pt.
19. randomized controlled trial.pt.
20. RANDOMIZATION/
21. random * allocat *.mp.
22. randomi?ed experimental trial *.mp.
23. controlled clinical trial/
24. controlled clinical trial *.mp.
25. clinical trial/
26. clinical trial *.mp.
27. clinical trial *.pt.
28. experimental trial *.mp.
29. single blind procedure/
30. double blind procedure/
31. triple blind procedure/
32. ((singl * or doubl * or tripl * or trebl *) adj25 (blind * or mask *)).mp.
33. (placebo * or (placebo * adj4 (trial * or control *))).mp.
34. or/15-33
35. 14 and 34

*Database*: HealthStar
*Date range*: 1966 to July 31, 2012
*Results*: 1170
1. advanced nursing practice *.mp.
2. advanced practice nurs *.mp.
3. APN.mp.
4. clinical nurse specialist *.mp.
5. (CNS and nurs *).mp.
6. nurse practitioners/
7. nurse practitioner *.mp.
8. (NP and nurs *).mp.
9. nurse specialist *.mp.
10. specialist nurse *.mp.
11. nurse clinicians/
12. nurse clinician *.mp.
13. nurse anesthetists/
14. nurse anesthetist *.mp.
15. nurse anaesthetist *.mp.
16. nurs * led.mp.
17. or/1-16
18. randomized controlled trials/
19. randomi?ed controlled trial *.mp.
20. randomized controlled trial.pt.
21. RCT *.mp.
22. random allocation/
23. random * allocat *.mp.
24. randomi?ed experimental trial *.mp.
25. controlled clinical trial *.mp.
26. controlled clinical trial.pt.
27. randomized controlled trial.pt.
28. controlled clinical trial *.mp.
29. experimental trial *.mp.
30. single-blind method/
31. double-blind method/
32. ((singl * or doubl * or tripl * or trebl *) adj25 (blind * or mask *)).mp.
33. (placebo * or (placebo * adj4 (trial * or control *))).mp.
34. or/18-33
35. 17 and 34

*Database*: Medline
*Date range*: 1950 to July 31, 2012
*Results*: 1349
1. advanced nursing practice *.mp.
2. advanced practice nurs *.mp.
3. APN.mp.
4. clinical nurse specialist *.mp.
5. (CNS and nurs *).mp.
6. nurse practitioners/
7. nurse practitioner *.mp.
8. (NP and nurs *).mp.
9. nurse specialist *.mp.
10. specialist nurse *.mp.
11. nurse clinicians/
12. nurse clinician *.mp.
13. nurse anesthetists/
14. nurse anaesthetist *.mp.
15. nurse anesthetist *.mp.
16. nurs * led.mp.
17. or/1-16
18. randomized controlled trial/
19. randomi?ed controlled trial *.mp.
20. RCT *.mp.
21. randomized controlled trials as topic/
22. randomized controlled trial.pt.
23. random allocation/
24. random * allocat *.mp.
25. randomi?ed experimental trial *.mp.
26. controlled clinical trial/
27. controlled clinical trial *.mp.
28. clinical trial.pt.
29. clinical trial *.mp.
30. experimental trial *.mp.
31. single-blind method/
32. double-blind method/
33. ((singl * or doubl * or tripl * or trebl *) adj25 (blind * or mask *)).mp.
34. (placebo * or (placebo * adj4 (trial * or control *)).mp.
35. or/18-34
36. 17 and 35

*Database*: AMED
*Date range*: all years to July 31, 2012
*Results*: 30
1. advanced nursing practice *.mp.
2. advanced practice nurs *.mp.
3. APN.mp.
4. clinical nurse specialist *.mp.
5. (CNS and nurse *).mp.
6. nurse practitioner *.mp.
7. (np and nurse *).mp.
8. nurse specialist *.mp.
9. specialist nurse *.mp.
10. nurse clinician *.mp.
11. nurse anesthetist *.mp.
12. nurse anaesthetist *.mp.
13. or/1-12
14. randomized controlled trials/
15. randomi?ed controlled trial *.mp.
16. RCT.mp.
17. randomized controlled trial.pt.
18. Random allocation/
19. random * allocat *.mp.
20. randomi?ation.mp.
21. randomi?ed experimental trial *.mp.
22. controlled clinical trial *.mp.
23. Clinical trials/
24. clinical trial *.mp.
25. clinical trial *.pt.
26. experimental trial *.mp.
27. ((singl * or doubl * or tripl * or trebl *) adj25 (blind * or mask *)).mp.
28. (placebo or (placebo * adj4 (trial * or control *))).mp.
29. or/14-28 *
30. 13 and 29
31. nurs * led.mp.
32. 31 or 13
33. 32 and 29

*Database*: Cochrane
*Date range*: all years to July 31, 2012
*Results*: 145
1. advanced nursing practice *.mp.
2. advanced practice nurs *.mp.
3. apn.mp.
4. clinical nurse specialist *.mp.
5. (CNS and nurse *).mp.
6. nurse practitioner *.mp.
7. (np and nurse *).mp.
8. nurse specialist *.mp.
9. specialist nurse *.mp.
10. nurse clinician *.mp.
11. nurse anaesthetist *.mp.
12. nurse anesthetist *.mp.
13. or/1-12
14. randomi?ed controlled trial *.mp.
15. RCT.mp.
16. random * allocat *.mp.
17. randomi?ed experimental trial *.mp.
18. controlled clinical trial *.mp.
19. clinical trial *.mp.
20. experimental trial *.mp.
21. ((singl * or doubl * or tripl * or trebl *) adj25 (blind * or mask *)).mp.
22. (placebo or (placebo * adj4 (trial * or control *))).mp.
23. or/14-22
24. 13 and 23
25. nurs * led.mp.
26. (13 or 25) and 23

*Database*: Cochrane Central
*Date range*: all years to July 31, 2012
*Results*: 474
1. advanced nursing practice *.mp.
2. advanced practice nurs *.mp.
3. apn.mp.
4. clinical nurse specialist *.mp.
5. (CNS and nurse *).mp.
6. nurse practitioner *.mp.
7. (np and nurse *).mp.
8. nurse specialist *.mp.
9. specialist nurse *.mp.
10. nurse clinician *.mp.
11. nurse anaesthetist *.mp.
12. nurse anesthetist *.mp.
13. or/1-12
14. randomi?ed controlled trial *.mp.
15. RCT.mp. *
16. random * allocat *.mp.
17. randomi?ed experimental trial *.mp.
18. controlled clinical trial *.mp.
19. clinical trial *.mp.
20. experimental trial *.mp.
21. ((singl * or doubl * or tripl * or trebl *) adj25 (blind * or mask *)).mp.
22. (placebo or (placebo * adj4 (trial * or control *))).mp.
23. or/14-22
24. 13 and 23
25. nurs * led.mp.
26. (13 or 25) and 23

*Database*: DARE
*Data range*: all years to July 31, 2012
*Results*: 85
1. advanced nursing practice *.mp.
2. advanced practice nurs *.mp.
3. apn.mp.
4. clinical nurse specialist *.mp.
5. (CNS and nurse *).mp.
6. nurse practitioner *.mp.
7. (np and nurse *).mp.
8. nurse specialist *.mp.
9. specialist nurse *.mp.
10. nurse clinician *.mp.
11. nurse anaesthetist *.mp.
12. nurse anesthetist *.mp.
13. or/1-12 *
14. randomi?ed controlled trial *.mp.
15. RCT.mp.
16. random * allocat *.mp.
17. randomi?ed experimental trial *.mp.
18. controlled clinical trial *.mp.
19. clinical trial *.mp.
20. experimental trial *.mp.
21. ((singl * or doubl * or tripl * or trebl *) adj25 (blind * or mask *)).mp.
22. (placebo or (placebo * adj4 (trial * or control *))).mp.
23. or/14-22
24. 13 and 23
25. nurs * led.mp.
26. (13 or 25) and 23

*Database*: HEED
*Data range*: all years to July 31, 2012
*Results*: 108
1. advanced nursing practice *.mp.
2. advanced practice nurs *.mp.
3. apn.mp.
4. clinical nurse specialist *.mp.
5. (CNS and nurse *).mp.
6. nurse practitioner *.mp.
7. (np and nurse *).mp.
8. nurse specialist *.mp.
9. specialist nurse *.mp.
10. nurse clinician *.mp.
11. nurse anaesthetist *.mp.
12. nurse anesthetist *.mp.
13. or/1-12
14. randomi?ed controlled trial *.mp.
15. RCT.mp.
16. random * allocat *.mp.
17. randomi?ed experimental trial *.mp.
18. controlled clinical trial *.mp.
19. clinical trial *.mp.
20. experimental trial *.mp.
21. ((singl * or doubl * or tripl * or trebl *) adj25 (blind * or mask *)).mp.
22. (placebo or (placebo * adj4 (trial * or control *))).mp.
23. or/14-22
24. 13 and 23
25. nurs * led.mp.
26. (13 or 25) and 23

*Database*: Web of Science
Includes: Science Citation Index, Social Sciences Index, Arts & Humanities Index, Conference Proceedings Citation Index (Science), Conference Proceedings Citation Index (Social Sciences and Humanities)
*Data range*: all years
*Results*: 1333
#16  #15 AND #8
#15  #14 OR #13 OR #12 OR #11 OR #9
#14 TS = (placebo * OR (placebo SAME (trial * OR control *)))
#13  TS = ((singl * or doubl * or tripl * or trebl *) AND (blind * OR mask *))
#12  TS = ((single or double or triple or trebl *) SAME blind procedure)
#11  TS = (clinical trial * OR experimental trial *)
#10  TS = ((randomi *ed controlled trial *) OR rct)
#9  TS = ((randomi *ed controlled trial *) OR rct OR randomi *ation OR random allocation OR randomi *ed experimental trial *)
#8  #7 OR #6
#7  TS =  (nurs * lead)
#6  #5 OR #4 OR #3 OR #2 OR #1
#5  TS = (nurse an *esthetist)
#4  TS = (nurse practitioner *)
#3  TS = (nurse * SAME specialist *)
#2  TS = ((apn or cns or np) AND nurse *)
#1  TS = (advanced SAME nurs * SAME practice *).
